# Preservation of high resolution protein structure by cryo-electron microscopy of vitreous sections

**DOI:** 10.1016/j.ultramic.2009.09.004

**Published:** 2009-12

**Authors:** Kasim Sader, Daniel Studer, Benoît Zuber, Helmut Gnaegi, John Trinick

**Affiliations:** a Institute of Molecular and Cellular Biology, University of Leeds, LS2 9JT, UK; b Institute for Materials Research, University of Leeds, LS2 9JT, UK; c SuperSTEM, J Block, Daresbury Laboratory, Warrington, Cheshire WA4 4AD, UK; d Institute of Anatomy, University of Bern, CH-3010, Switzerland; e MRC Laboratory of Molecular Biology, Cambridge CB2 0QH, UK; f Diatome, Biel, Switzerland

**Keywords:** Cryo-section, Section resolution

## Abstract

We have quantitated the degree of structural preservation in cryo-sections of a vitrified biological specimen. Previous studies have used sections of periodic specimens to assess the resolution present, but preservation before sectioning was not assessed and so the damage due particularly to cutting was not clear. In this study large single crystals of lysozyme were vitrified and from these X-ray diffraction patterns extending to better than 2.1 Å were obtained. The crystals were high pressure frozen in 30% dextran, and cryo-sectioned using a diamond knife. In the best case, preservation to a resolution of 7.9 Å was shown by electron diffraction, the first observation of sub-nanometre structural preservation in a vitreous section.

## 1 Introduction

Determination of the structure of intact biological tissues and cells to the highest possible resolution is an important goal across biology and medicine, and transmission electron microscopy (TEM) remains the most powerful and widely used technique available for this purpose. However, the routinely achieved resolution in chemically fixed and resin-embedded samples is usually only ∼50 Å, which is well below what is needed to recognise the shapes of most protein molecules. This precludes easy integration of higher resolution EM, X-ray or NMR component structures into the cellular context, which is a major barrier to understand the cell structure and mechanism.

Cryo-sectioning of vitrified tissue avoids the chemical treatments involved in plastic embedding and sectioning; it is therefore thought to achieve better preservation and avoid the artefacts of chemical treatments [1,2]. However, decades after its inception, vitreous cryo-sectioning is still regarded as a difficult technique and is routine only in specialist labs. Moreover, it is accompanied by severe compression (15–35% [3]) in the cutting direction and there have been few quantitative studies of what structural preservation can be attained with the method.

Preservation is most easily assessed from periodic structure, using Fourier transforms of images or by electron diffraction. The best resolution reported was by Richter [4] who demonstrated 30 Å detail in cryo-sections of catalase crystals and 22 Å detail in hexagonal DNA crystals, whilst Blanc et al. [5] demonstrated 27 Å detail in DNA from stallion sperm. This discrepancy between what is assumed to be the near-perfect initial preservation of vitrified samples and the much lower resolution seen in cryo-sections suggests that considerable damage is caused by the process of cutting. On the other hand, observations of higher resolution features (better than 20 Å) have been (routinely) observed in projection images of cross-sectioned microtubules [6], although resolution could not be precisely measured since the features were not crystalline.

In the case of plastic-embedded specimens, there have been a few studies where the resolution of the sample was first assessed by X-ray diffraction before sectioning in order to monitor preservation at different stages during embedding. These studies generally showed high resolution detail (up to ∼1 nm) in the uncut block, but much worse preservation in the subsequent sections, suggesting that cutting is a major source of damage [7–9]. Moreover, a recent X-ray diffraction study demonstrated reflections to better than 6 Å from crystals of lysozyme embedded in plastic, thus showing that embedded protein can be very well preserved and again consistent with the idea of damage due to cutting [10].

However, no comparable assessment has been done for specimen preservation before cryo-sectioning, and so it was not clear whether the limited resolution reported by diffraction or Fourier analysis of images was due to damage occurring during freezing, sectioning and/or imaging, or whether the crystals were partially disordered. In this paper we report on the preservation of frozen lysozyme crystals, crystallised under conditions that allowed X-ray diffraction to better than 2.1 Å resolution, and of which electron diffraction of cryo-sections gave reflections extending to 7.9 Å. The data therefore shows sub-nanometre preservation in a vitreous section for the first time.

## 2 Materials and methods

### 2.1 Lysozyme crystal preparation

Hen egg-white lysozyme (Sigma L-6876) at 40 mg/ml was crystallised by the sitting drop method in a solution of 10 mM citrate buffer [11] with 1.1 M NaCl at pH 4.7. Blue dye (1% SuperCook Blue containing Brilliant Blue FCF) was added to the crystallisation droplet for the crystals used for cryo-sectioning in order to facilitate locating the crystal during manipulation and sectioning.

### 2.2 X-ray diffraction

Crystals were removed from the crystallisation wells by pipette tip, an equal volume of 30% glycerol added, and then transferred to fresh 30% glycerol with a nylon loop. Within 30 s these were vitrified by freezing in liquid nitrogen, or a stream of cold dry nitrogen gas (100 K, Oxford Cryosystems) attached to a Rigaku RU200 rotating anode X-ray generator (Rigaku Corp., Woodland, TX).

The frozen lysozyme crystals were mounted in a 100 K dry nitrogen stream. They were visually evaluated for ice-crystal formation and crystal morphology where possible, and centred for the X-ray beam using a stereoscope. Data were recorded with a laboratory Raxis IV image plate detector. X-ray radiation of 1.542 Å wavelength (CuK*α*) was used.

Single exposures were taken of all crystals with an oscillation angle of 1.5° and an exposure time of 10 min. The distance between the crystal and the X-ray detector was 160 mm, giving a resolution of 2.1 Å for X-rays scattered to the edge of the detector. All data processing, including auto-indexing, was performed using MOSFLM [12].

### 2.3 Cryo-sectioning

Tetragonal lysozyme crystals with a long [001] (*c*) axis from separate crystallisations were transferred to a droplet of 30% dextran–PBS ensuring that visible dehydration did not occur. The droplet was mixed thoroughly to remove any residual crystallisation buffer around the crystal. Good crystals appeared stable in the dextran solution over a period of minutes. The crystals were transferred to a small 30% dextran droplet on a 200-μm-deep membrane carrier (Leica) for high-pressure freezing with a Leica EMPACT II. Membrane carriers were examined for signs of expansion on the side opposite to the droplet, which was assumed to be a sign of imperfect vitrification, and those with expansion discarded. This is an empirical observation, but has the likely explanation that when water crystallises, its density decreases and therefore it expands and deforms the carrier. This is important because cryo-sectioning can transform crystalline into amorphous ice [13].

Membrane carriers were orientated so that the [001] axis of the lysozyme crystal was perpendicular to the cutting direction. Trimming was performed with a 45° cryotrim diamond knife (Diatome) at 100 mm/s with a 200 nm feed for the metal membrane carrier and 50 nm feed once in the vitrified dextran. Cutting was performed with a 35° cryo-immuno diamond knife (Diatome) at −140 °C and ribbons transferred to Quantifoil 7/2 square mesh grids covered with a 2 nm amorphous carbon film [14].

### 2.4 Electron diffraction

Low-dose electron diffraction was performed on a Tecnai F30 microscope at 200 kV with a Gatan 626 cryoholder. The sections were located in low magnification mode, following which the area was viewed using a highly defocussed diffraction pattern. A 20 μm C2 aperture and spot size 6 was used with low C2 excitation to illuminate a large area. Once an area of interest was located, C2 was adjusted to define only that area, and the diffraction pattern focussed. The diffraction patterns were recorded on a 2k TVIPS F224 CCD camera with 24 μm pixels. Camera lengths were calibrated with catalase platelets (Agar) using an *a* unit cell length of 69 Å [15,16]. The signal-to-noise ratio (SNR) of the diffraction spots was calculated by subtracting the background level from the peak height, and dividing by the standard deviation of the background.

## 3 Results and discussion

### 3.1 Lysozyme crystallisation and X-ray diffraction

**Fig. 1:**
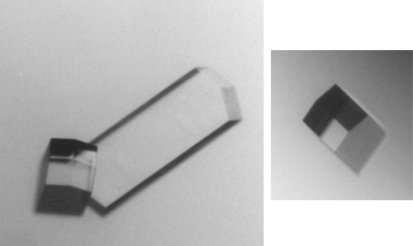
Tetragonal lysozyme crystals. The long axis [001] of the large crystal on the left is greater than 500 μm.

**Fig. 2:**
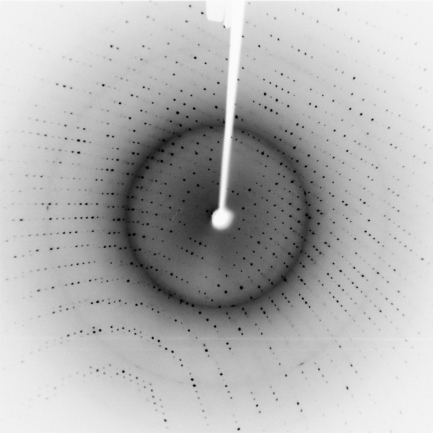
X-ray diffraction pattern from lysozyme crystal showing better than 2.1 Å resolution.

Large lysozyme crystals (>0.5 mm) were grown in one week and the shape of these was typical of the tetragonal form (Fig. 1). All of the glycerol-embedded crystals diffracted X-rays to better than 2.1 Å, an example of which is shown in Fig. 2. The lysozyme space group determined by MOSFLM [12] was primitive tetragonal (P4) with unit cell dimensions of *a*=*b*=79.03 Å, *c*=37.04 Å. This diffraction pattern is representative of the average quality of more than 12 that were collected from different crystals.

### 3.2 Cryo-sectioning

In initial experiments the glycerol served as a first embedding medium in addition to its function as a cryo-protectant, and allowed manipulation of the crystals. The glycerol-embedded crystals were remounted with cryo glue (2:3 ethanol:2-propanol [17]) for cryo-sectioning. However, the sectioning properties of glycerol/cryoglue were not optimal. Glycerol increases the formation of crevasses, and biological specimens in glycerol tend to be brittle [18], while cryoglue makes it more difficult to see through the ice and find the specimen. It can also stick to the knife and cause problems during sectioning. The remounting process of the glycerol-embedded crystal into the cryoglue itself introduced a step for possible crystal damage. Despite these problems, some sections were obtained. Preliminary electron diffraction data from cryo-sections of glycerol-embedded crystals suggested that better than 10 Å structural preservation would be possible.

The crystal preparation process was optimised using blue dye for crystal visibility, and high pressure freezing with 30% dextran for reproducible freezing in a form suitable for cryo-sectioning. Lysozyme that was crystallised in the presence of similar concentrations of the same dye routinely diffracted to better than 2 Å (Miroslav Papiz, private comm.). High pressure freezing in dextran in a membrane carrier was a much more reproducible method of producing visibly well-frozen crystals that retained sharp edges and did not crack. It would be interesting to study the effects of high pressure freezing in dextran on the lysozyme crystal structure by X-ray diffraction, but this was not done in this study. The sectioning properties of the dextran-embedded crystals were good, with no crevasses or knife chatter. Dextran has a high molecular weight (∼40 kDa) and the vitrified solution may act more as a gel and therefore be less brittle than glycerol, preventing crevasse formation.

### 3.3 Electron diffraction of dextran-embedded lysozyme

**Fig. 3:**
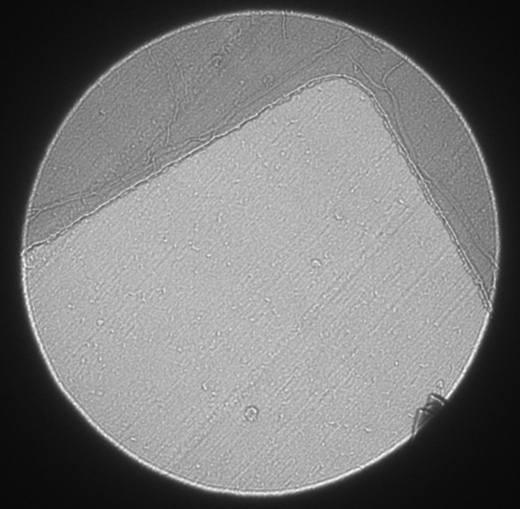
Defocussed electron diffraction pattern showing the area of a vitreous cryo-section of lysozyme from which diffraction patterns in Figs. 4 and 5 were recorded. The denser L-shaped area corresponds to the edge of the supporting square mesh.

**Fig. 4:**
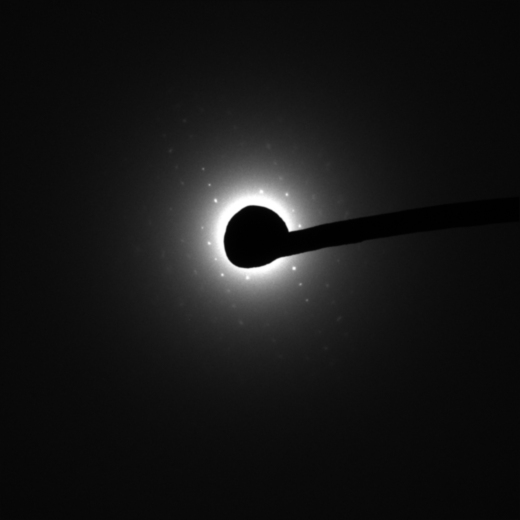
Electron diffraction pattern from 2 s exposure at 4.5 m camera length with brightness and contrast adjusted so that low resolution reflections are visible.

**Fig. 5:**
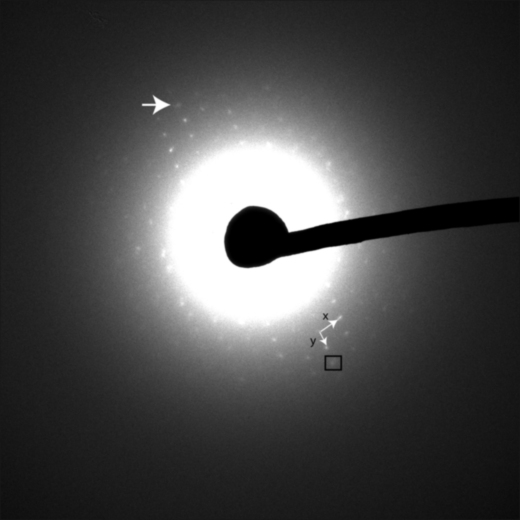
Electron diffraction pattern from 5 s exposure at 4.5 m camera length with brightness and contrast adjusted so that the highest resolution reflections are visible. Large arrow (top) points to reflection at 7.9 Å. The Friedel-related reflection is enclosed by a box (bottom). Lattice parameters *x* (51 Å) and *y* (70 Å) are indicated by small arrows.

Electron diffraction patterns were recorded from the area shown in the defocussed diffraction pattern in Fig. 3, which is approximately 5 μm in diameter. Focussed diffraction patterns were recorded with exposure times of 2 and 5 s at the calibrated camera length of 4.5 m and are shown displayed with different contrast levels in Figs. 4 and 5. The first diffuse diffraction ring from vitreous water was observed with shorter camera lengths, and no reflections from crystalline ice. In Fig. 5, the furthest diffraction spot with a SNR>5 lies at 593 pixels from the undiffracted beam, corresponding to 7.9 Å. Of the four best electron diffraction patterns from different sections (from three ribbons on two grids), the mean resolution was 9.0±1.3 Å.

This resolution is nearly three-fold better than the highest resolution data from a cryo-sectioned crystalline specimen in the literature of 22 Å by Richter [4]. This can be explained in that Richter did not assess the preservation of the specimens, except that of catalse by negative staining, which does not generally preserve the structure to greater than 20 Å. Also, high-pressure freezing was not used. Overall there have been improvements in equipment, diamond knives, and techniques, but the choice of the well-ordered specimen of lysozyme is probably most important. Practical improvements to lysozyme preparation are still possible. While the dye solved the problem of observing the crystal during preparation, it was difficult to identify the crystal from the dextran in the sections in the electron microscope as they both are of similar densities. A possible strategy to overcome this would be to coat the crystal surface with colloidal gold, which binds non-specifically to proteins [19]. This could allow the identification of the sectioned protein crystal and facilitate systematic experiments with lysozyme crystals.

The diffraction spots in Fig. 5 were manually indexed, giving lattice parameters of 51 Å (*x*) and 70 Å (*y*). These values are compatible with the unit cell dimensions determined from the X-ray diffraction patterns by MOSFLM (*a*=*b*=79 Å), as expected since the crystal was cut in this orientation. However, they are not equal or orthogonal, suggesting deformation, most likely because of section compression. There is no rotation focussing the diffraction pattern, and therefore the low-resolution image formed by the defocussed diffraction pattern can be directly compared to the focussed diffraction pattern. The direction of cut visible from the knife marks in Fig. 3 is roughly parallel to the *x* lattice spacing of the diffraction pattern. Compression could have brought the *x* lattice planes closer together and therefore reduced the *x* lattice spacing. This would explain the smaller *x* lattice spacing compared to *y*. Further experiments could completely describe the deformation by electron diffraction of tilted sections, determining the third unit cell dimension.

The main advantage of using electron diffraction to assess preservation is that extremely low doses can be used compared to imaging. This is possible because there is no contrast transfer function envelope or modulation transfer function of the detector to degrade the high-resolution information. Also, diffraction patterns are insensitive to specimen drift. The doses used here are estimated to be well below 1 e^−^/Å^2^. The diffraction patterns were not observed to change over several exposures at different camera lengths, also suggesting that radiation damage was not an issue.

However, if images of similar sections were recorded there is every expectation that similar power spectra could be obtained. The envelope function of the contrast transfer function is not as large a factor at 8 Å as at 4 Å. It is possible to record an image with a 2k×2k camera so that 8 Å is below 0.5 of the Nyquist frequency, where the modulation transfer function of the 2 k TVIPS 224 CCD is about 0.5 (at 120 kV) [20]. Under these conditions the sample area imaged would only be 0.16 μm^2^ compared to the ∼19 μm^2^ sampled for electron diffraction here, reducing the SNR of the diffraction spots in the Fourier transform. Film would be a better choice as it can record over 10k×10k pixels of 5–10 μm [21] with a similar value of the modulation transfer function at 0.5 of the Nyquist frequency at 120 kV and better at 300 kV [20], allowing up to 4 μm^2^ to be recorded at a sampling rate of 2 Å/pixel. This could allow the procedure of real space unbending to be applied, which has been widely used in 2D electron crystallography [22,23]. Real space unbending was devised to correct for lattice disorder in 2D crystals; it produces a map of vector displacements of distortion in crystal images. The distortions are removed by interpolation, giving significant resolution improvement in Fourier transforms. This could be used to correlate deformations found in the crystal lattice with sectioning artefacts at high resolution.

## 4 Conclusion

Cryo-sectioning has the potential advantage that vitrified samples can be ideally preserved before cutting. However, cryo-sectioning is technically difficult and the resolution attainable has not been extensively evaluated. Our results from sectioning experiments of lysozyme show high-resolution diffraction to 7.9 Å, the first demonstration of sub-nanometre resolution in an ultramicrotome section of a biological specimen. It therefore seems possible that tomography of cryo-sections could be extended to the theoretical limit of ∼20 Å proposed for this technique, based on signal-to-noise considerations [24,25], and that 3D averaging could be used to surpass this limit. Vitrified lysozyme crystals could also be used as a test specimen for use in minimizing cryo-sectioning artefacts affecting preservation of high resolution detail. Systematic tests of different sectioning conditions including knife angles, feeds, speeds, and temperatures could now be undertaken with a quantitative measure of resolution.
